# Backward- and Forward-Looking Potential of Anaphors

**DOI:** 10.3389/fpsyg.2015.01746

**Published:** 2015-11-23

**Authors:** Petra B. Schumacher, Jana Backhaus, Manuel Dangl

**Affiliations:** Department of German Language and Literature I, University of CologneCologne, Germany

**Keywords:** pronoun resolution, prominence, agentivity, position, ERP, N400, Late Positivity, topic shift

## Abstract

Personal pronouns and demonstratives contribute differently to the encoding of information in the mental model and they serve distinct backward- and forward-looking functions. While (unstressed) personal pronouns are the default means to indicate coreference with the most prominent discourse entity (backward-looking function) and typically mark the maintenance of the current topic, demonstratives are used to refer to a less prominent entity and serve the additional forward-looking function of signaling a possible topic shift. In Experiment 1, we present an ERP study that examines the time course of processing personal and d-pronouns in German (*er* vs. *der*) and assesses the impact of two prominence features of the antecedent, thematic role and sentential position, as well as neurophysiological correlates of backward- and forward-looking functions of referential expressions. We tested the comprehension of personal and d-pronouns following context sentences containing two potential antecedents. In addition to the factor pronoun type (*er* vs. *der*), we varied the verb type (active accusative verbs vs. dative experiencer verbs) and the thematic role order (canonical vs. non-canonical) in the context sentences to vary the antecedent's prominence. Time-locked to pronoun-onset, the ERPs revealed a general biphasic N400-Late Positivity for d-pronouns over personal pronouns with further subtle interactions of the prominence-lending cues in the early time window. The findings indicate that the calculation of the referential candidates' prominence (backward-looking function) is guided by thematic role and positional information. Thematic role information, in combination with initial position, thus represents a central predictor during referential processing. Coreference with a less prominent entity (assumed for d-pronouns) results in processing costs (N400). The additional topic shift signaled by d-pronouns (forward-looking function) results in attentional reorienting (Late Positivity). This is further supported by Experiment 2, a story continuation study, which showed that personal pronouns trigger topic maintenance, while d-pronouns yield topic shifts.

## Introduction

When a language makes available different forms to refer to entities in the world, these forms typically indicate discrete cognitive states in the mental representation of the interlocutors (cf. Gundel et al., 1993). Accordingly, personal pronouns, demonstrative pronouns, definite noun phrases (NPs) or indefinite NPs serve distinct discourse pragmatic functions. In the following, we will focus on the contribution of personal and demonstrative pronouns to reference tracking. While (unstressed) personal pronouns are the default means to indicate coreference with the most prominent entity in the current discourse, demonstrative pronouns are used to refer to a less prominent entity or exclude the most prominent entity (cf. Comrie, 1997). We refer to this as the “backward-looking function” of referential expressions. In addition, personal pronouns signal the maintenance of the current topic, while demonstratives suggest that the respective referent is likely to be promoted to topic status in subsequent discourse and thus indicate a topic shift (cf. e.g., Abraham, 2002). This is what we call the “forward-looking function.”

Demonstratives come in pronominal (*this, that*) or adnominal form (*this teacher, that book*) and represent deictic expressions that mark the relative distance of the respective referent to the speaker, the hearer or both. Languages vary with regard to how many distance contrasts they encode and whether they only consider the speaker as the deictic center or allow for perspectival centers associated with other protagonists as well; for example English distinguishes the near *this* and the distant *that*, Spanish has a three-way contrast (proximal: *este*, medial: *ese*, distal: *aquel*), Hausa a four-way contrast (near speaker: *nân*, near hearer: *nan*, away from speaker and hearer: *cân*, far away from speaker and hearer: *can*), and some systems encode even more contrasts (e.g., Navajo, Malagasy; Diessel, 2013). German, the language under investigation in this study, employs the demonstrative pronouns *dieser, diese, dieses* (masculine, feminine, neuter) and the d-pronoun *der, die, das*. The former is more restricted in its referential choice and is claimed to prefer the last mentioned entity as its referential candidate, while the d-pronoun does not have such a local restriction (cf. e.g., Zifonun et al., 1997). A less commonly used form to mark distance is *jener, jene, jenes*, but German more frequently uses a modifying adverbial (*hier* “here,” *da* “there”) to mark distance contrasts.

In the current investigation, we compare the comprehension of the d-pronoun *der* with that of the personal pronoun *er* in contexts with two potential antecedents. The resolution preferences are generally discussed with reference to the notion of referential prominence, which assumes that referents that are accessible in the mental model are ranked in a particular order (cf. e.g., Grosz et al., 1995). But what is prominence? In the literature on pronoun resolution many different factors have been discussed as prominence-lending cues and in the following we provide a brief overview over possible candidate features assumed in the processing literature.

The most influential accounts that investigated personal and demonstrative pronoun resolution considered syntactic function and topicality to be prominence-lending features. Bosch and colleagues initially proposed that personal pronouns in German show a subject preference, while d-pronouns have an anti-subject preference (Bosch et al., 2003). Based on examples with clear discourse topics, they subsequently suggest that personal pronouns favor topical entities and d-pronouns follow an anti-topic interpretation strategy (Bosch and Umbach, 2007; Hinterwimmer, 2015). These accounts assume complementary interpretation preferences for personal and d-pronouns. By contrast, on the basis of data from Finnish, where the personal pronoun was preferably interpreted to refer to the subject while the demonstrative elicited a last-mention preference, Kaiser proposed a non-complementary form-specific distribution of interpretation preferences (Kaiser and Trueswell, 2008). Research on pronoun resolution has identified numerous other candidate factors, including among others linear order, animacy, focus, coherence relations and verb semantics (Stevenson et al., 1994; Chambers and Smyth, 1998; Järvikivi et al., 2005; Kehler et al., 2008; Ellert, 2010).

An alternative account of pronoun resolution is the Bayesian model which promotes a tight relationship between pronoun interpretation and production (Kehler et al., 2008; Kehler and Rohde, 2013). In this framework, interpretive preferences are not merely a function of the prominence structure of previous discourse but arise from the combination of prior expectations for subsequent mention and the production bias for a particular form. Behavioral research within this framework suggests that grammatical function or topichood influence the production bias while coherence relations impact which referent is expected. This approach thus assumes that prominence-lending cues feed into an intricate system of predictive processing that shapes expectation for a particular referent and considers production biases for a particular form. This line of research is promising, but in the current research we do not tease apart production biases and prior expectation. We assess the mechanisms underlying pronoun processing but future research should follow up on the Bayesian predictions within our experimental design.

The current research asks the question whether thematic function is a high ranked candidate for referential prominence. This is motivated by claims that agentivity is part of core cognitive architecture and shapes our thinking and cognitive development in fundamental ways (Leslie, 1995). According to this view, agents are cognitive attractors that hold certain causal properties, initiate actions, pursue goals, have sentience. This is reminiscent of the feature-based characterization of agentivity in semantic theories that attributes causation, volitionality, sentience, self-propelled movement and independent existence to prototypical agents (Dowty, 1991; Primus, 1999). These theories have proposed thematic role hierarchies on the basis of proto-roles, with the highest thematic role being the “proto-agent” and the lower one the “proto-patient.” According to this view, agents are the prototypical exemplar of proto-agent because they hold many of the properties listed above but experiencers also satisfy features of proto-agents. Previous research on pronoun resolution has already pointed to the contribution of thematic role information by looking at verb semantics and animacy, and subject or topic preferences may be explained by agent preferences as well, since these features are often aligned.

To disentangle the effect of thematic role from grammatical function, we investigate reference resolution in the context of antecedent clauses with dative experiencer verbs, which critically cross these two predictors for prominence and have an agentive object (i.e., the experiencer) and a non-agentive subject. Example (2) illustrates this construction. In this example, the boxer is the experiencer and the one who must be sentient based on possible verbal entailments about the argument; hence the object holds more proto-agent properties than the subject.

1. Der Feuerwehrmann will      den Jungen       retten … Aber er/der hat …
  The firefighter-NOM wants the boy-ACC rescue … But he/D-Pro has …
  “The firefighter wants to rescue the boy … But he has… ”
2. Dem Boxer      hat der Musiker       imponiert … Aber er/der hat …
  The boxer-DAT has the musician-NOM impressed …. But he/D-Pro has …
  “The boxer was impressed by the musician … But he has … ”

Different prominence-lending features of the referents introduced in the context sentences may be responsible for pronoun resolution preferences in these two examples. Crucially, the context sentences differ with respect to the adherence and alignment of the following prominence cues: (i) agentivity (proto-agent > proto-patient), (ii) grammatical function (subject > object) and (iii) topicality. Note that for topicality we assume that the initial argument of a sentence represents the aboutness-topic (cf. Reinhart, 1981). Thus, rather than considering first vs. second mention effects, we pursue a functional approach according to which first mentioned referents serve as topics. Table 1 illustrates the prominence-lending features incorporated by the initial argument in the active accusative context (1) and the dative experiencer context (2) for canonical and non-canonical argument order. The possible candidate features agent, subject and topic are fully aligned in the canonical active accusative case. The dative experiencer conditions represent an alignment of two of these features and will help to disentangle the contribution of agentivity and subjecthood. Finally, the non-canonical active accusative condition shows even less alignment at the initial argument. If harmonic alignment at the initial position is a key to pronoun resolution, this condition should yield less clear preferences.

**Table 1:** **Prominence features of first argument in context sentence**.

	**Active accusative verbs**	**Dative experiencer verbs**
Canonical order	Agent and Subject and Topic	Agent and Topic
Non-canonical order	Topic	Subject and Topic

As an alternative to alignment of prominence-lending cues, one feature or a combination of features may affect pronoun resolution. For instance if thematic function is a decisive feature during pronoun resolution, this may be reflected in interpretive preferences irrespective of verb type and canonicity. If two or more features act jointly, fine differences should be observable when testing different verb types and canonicity effects. For example, if agentivity and topicality act together, the pronoun following the canonical dative-experiencer construction should link with its antecedent more easily than that following the non-canonical dative-experiencer context; if subjecthood and topicality collaborate, the non-canonical dative-experiencer antecedent clauses should yield clearer interpretive preferences than the canonical dative-experiencer contexts; etc.

Previous behavioral studies indicate a combination of partial feature alignment and the role of thematic function information. In offline tasks, agentivity has been shown to be a stronger predictor than subjecthood for pronoun resolution in German (Schumacher et al., 2016). Sentence completion and referent identification tasks with stimuli that contained either an antecedent clause with active accusative verbs [“rescue” in (1) where topic, subject and agent are aligned] or dative experiencer verbs [“be impressed” in (2) where the proto-agent, the xperiencer, is the object] revealed a proto-agent bias for the personal pronoun and an anti-agent bias for the d-pronoun in the canonical argument order of (1) and (2). When the argument order in the context clause was reversed, the active accusative verbs still registered an agent (or subject) preference—contra first mention or topic preference accounts of personal pronoun resolution—and an anti-agent (anti-subject) bias for the d-pronoun. Argument reversal of (2) resulted in chance performance for both types of pronouns suggesting that in this case the calculation of the relative ranking of the referential candidates was hampered. These data indicate that in a task in which participants are not under time pressure agentivity outweighs subjecthood when it is aligned with topic and/or subject—i.e., in the canonical accusatives (where all three cues are aligned), the canonical dative experiencers (where agent and topic are aligned), and the non-canonical accusatives (where agent and subject are aligned). This suggests that alignment of certain prominence-lending features is beneficial for pronoun resolution. In the case where the agent is not aligned with either topic or subject (the non-canonical dative experiencer contexts), the relative ranking of the referents seems to be too weak to generate an interpretive preference for either of the referential candidates. This reveals that interpretive preferences are not just a consequence of (partial) alignment of prominence-lending cues but that the weighting of these cues is also of relevance.

In the current research, Experiment 1 was designed to investigate the real-time consequences of the verb type × canonicity manipulation for pronoun resolution through event-related brain potentials (ERPs). We hypothesize that prominence-lending cues are used for the generation of fine-tuned predictions about upcoming entities. Personal pronoun resolution as a potential means to signal topic maintenance may thus proceed relatively effortless but could be encumbered in cases in which prominence cues are difficult to process, for example due to certain types of misalignment (as illustrated in Table 1 and by the behavioral data). D-pronouns in turn require the exclusion of the most prominent referential candidate, which should result in processing costs. Based on previous ERP research, prediction errors—here assumed to be guided by prominence cues—should be reflected in a negative brain potential (N400; for an overview see Bornkessel-Schlesewsky and Schumacher, 2016). N400 effects have for instance been observed for referents of differing degrees of givenness—with given entities being more predictable than inferrables and new entities being the least expected (Burkhardt, 2006)—or as an indicator of the distance between anaphor and antecedent—with effects of first mention and recency across multiple sentences (Streb et al., 2004). Negative deflections have also been reported for referential ambiguity during pronoun resolution, which may indicate that a disambiguating referential form is expected in such cases (Nieuwland and Van Berkum, 2006).

With regard to the forward-looking function of demonstrative pronouns, psycholinguistic investigations have been sparse. It has been claimed that demonstratives have the potential to initiate a topic shift and promote their referent to topic status in later discourse. For example, Abraham (2002) explicitly describes the demonstrative as a topic shifter. Empirical evidence comes from the comparison of indefinite *this* (“this egg”) and regular indefinites (“an egg”; cf. e.g., Gernsbacher and Shroyer, 1989; Chiriacescu, 2011). Using text continuation tasks, in which participants were instructed to continue a story with five sentences, these studies found that indefinite *this* elicited more mentions of the referent in the continuations, with less marked forms, and had a higher topic shift potential than the regular indefinite. The function of a demonstrative is thus not only to draw the attention to a less prominent discourse entity but also to signal the comprehender that the respective referent may become more prominent in subsequent discourse. Experiment 2 was conducted to investigate the topic shift potential of d-pronouns (and topic maintenance potential of personal pronouns) using a text continuation task. The assumed shift in attention furthermore is predicted to have consequences for discourse representation. Previous research on Japanese and Chinese, in which the notion of topic is crucial for sentence processing, suggests that topic-marked entities that trigger a shift in the ranking of discourse referents and hence require the updating of discourse representation structure evoke a Late Positivity (Hirotani and Schumacher, 2011; Hung and Schumacher, 2012, 2014; Wang and Schumacher, 2013). We therefore predict a Late Positivity for discourse updating due to the topic shift potential of d-pronouns in Experiment 1.

## Experiment 1

The current experiment was designed to assess the online processing of d-pronouns and personal pronouns with a particular focus on contexts in which subject and agent were not aligned. We therefore tested active accusative and dative experiencer antecedent clauses with canonical and non-canonical argument order (see Table 2 for sample stimuli). As described above, dative experiencer constructions were chosen because they allow us to disentangle the contribution of thematic and syntactic function to pronoun resolution. These verbs come with a dative experiencer (proto-agent in the frameworks of Dowty, 1991 and Primus, 1999) and a subject that represents the lower ranked role and have already shown robust effects of agentivity in behavioral tasks (Schumacher et al., 2016). Note also that we assume that the canonical argument order for these constructions is object before subject (cf. e.g., Haider, 1993; but see Footnote 1 in the Discussion for an alternative view).

**Table 2:** **Example stimuli for the ERP experiment**.

**Argument order**	**Sentence**	**Stimuli**
**VERB TYPE: ACCUSATIVE VERB**		
Canonical	Context sentence	Der Feuerwehrmann | will | den Jungen | retten, | weil | das Haus | brennt. The firefighter-NOM wants the boy-ACC rescue because the house-NOM burns. *The firefighter wants to rescue the boy, because the house is burning*.
	Target sentence	Aber | **er/der** | ist | viel | zu | aufgeregt. But he/D-Pro is way too nervous. *But he is way too nervous*.
Non-canoncial	Context sentence	Den Jungen | will | der Feuerwehrmann | retten, | weil | das Haus | brennt. The boy-ACC wants the firefighter-NOM rescue because the house burns. *The firefighter wants to rescue the boy, because the house is burning*.
	Target sentence	Aber | **er/der** | ist | viel | zu | aufgeregt. But he/D-Pro is way too nervous. *But he is way too nervous*.
Verification Question	Correct answer “Yes”	Brennt das Haus? *Is the house burning?*
	Correct answer “No”	Wackelt das Haus? *Is the house shaking?*
**VERB TYPE: DATIVE EXPERIENCER VERB**		
Canonical	Context sentence	Dem Boxer | hat | der Musiker | imponiert, | und | zwar | schon | lange. The boxer-DAT has the musician-NOM impressed, in fact already long. *The boxer was impressed by the musician for a long time*.
	Target sentence	Aber | **er/der** | wollte | das | nicht | wahr | haben. But he/D-Pro wanted that not true have. *But he didn't want to accept it*.
Non-Canonical	Context sentence	Der Musiker | hat | dem Boxer | imponiert, | und | zwar | schon | lange. The musician-NOM has the boxer-DAT impressed, in fact already long. *The boxer was impressed by the musician for a long time*.
	Target sentence	Aber | **er/der** | wollte | das | nicht | wahr | haben. But he/D-Pro wanted that not true have. *But he didn't want to accept it*.
Verification question	Correct answer “Yes”	Imponierte der Musiker dem Boxer? *Has the musician impressed the boxer?*
	Correct answer “No”	Imponierte der Musiker dem Fechter? *Has the musician impressed the fencer?*

Concerning backward-looking, the core function of a pronoun is to refer to an entity available in the mental representation. Hence upon encountering a pronominal expression, a dependency relation between the pronoun and its antecedent must be established. This is guided by the prominence structure of the referents from prior discourse, resulting in a ranked set of referential candidates. Accessibility theories suggest that the personal pronoun prefers the most prominent entity or the entity in focus, which has been attested by corpus research and psycholinguistic experiments (cf. e.g., Gordon et al., 1993; Gundel et al., 1993). Accordingly, personal pronoun resolution should generally proceed rather effortlessly. By contrast, resolution of the d-pronoun has been described to exclude the highest ranked referential candidate (cf. Comrie, 1997; Abraham, 2002). Such an operation should be resource-consuming. All other things being equal, processing the d-pronoun should thus be more costly than processing the personal pronoun. With respect to ERP signatures, we hypothesize that the backward-looking function is first of all closely tied to this form-function correlation interacting with predictive referential parsing reflected in an N400 effect. For predictive parsing, the d-pronoun as the more marked form should be generally more costly than the personal pronoun because it requires the exclusion of the most prominent referent.

This process may be further affected by the misalignment or weighting of prominence features that may encumber the establishment of a ranked set of referential candidates. The experimental design allows us to investigate the organization of the possible set of prominence-lending features and its impact on real-time processing. We thus predict subtle interactions of the factors verb type (varying the combination of grammatical and thematic roles) and canonicity (assigning different topics) on pronoun resolution. If alignment of topic, subject and/or agent is a key force during online pronoun resolution, the different alignments illustrated in Table 1 may result in processing effort reflected by the N400 amplitude. Likewise the weighting of the different prominence-lending features may affect the processes underlying the N400.

With regard to the forward-looking function, the literature assumes that d-pronouns are topic shifters, which we argue has consequences for discourse updating. We therefore expect a Late Positivity effect for the d-pronoun relative to the personal pronoun. Previous research has not considered the role of prominence cues on forward-looking processes but misalignment of prominence features may result in failure to rank the referential candidates, which may well encumber forward-oriented processing.

### Methods

#### Participants

Twenty-seven right-handed, monolingually raised native speakers of German (14 women; mean age: 22; range 19–32) from the University of Mainz participated in this study after giving written informed consent. Participants had normal or corrected-to-normal vision and had no history of neurological or psychiatric disorders. The study was performed in accordance with the Declaration of Helsinki and with the national and institutional recommendations of the Neurolinguistics Lab at the Johannes Gutenberg-University Mainz. Data from three candidates were excluded from the ERP analysis due to excessive artifacts.

#### Materials

Sample stimuli for the eight conditions can be found in Table 1. The first sentence included two NPs that were masculine, animate and definite. In the accusative contexts, the canonical argument order was subject–object, and in the dative experiencer contexts, it was object–subject. Each of the context sentences was followed by a subordinate clause, which contained at most one gender-incongruent referent, to ensure that there was a proper distance between the NPs and the critical pronoun. The target sentence was always introduced by “but,” followed by either the personal pronoun “er” or the d-pronoun “der.” Sentence completions were kept referentially ambiguous. The material consisted of 60 accusative sets and 60 dative experiencer sets. Additionally, 60 filler sentence pairs were constructed, which included a masculine and feminine antecedent thus eliminating the ambiguity of the pronoun. Each participant was presented with 300 quasi-randomized test items: 240 critical items, consisting of 120 sentences with accusative verb and 120 with dative-experiencer verb, and all 60 fillers. Comprehension questions for each item served to assure that participants were paying attention to the stimuli. Correct and incorrect responses were evenly distributed across the stimuli. The incorrect comprehension questions targeted either an NP from the main clause, the action of the main clause or an element in the subordinate clause of the context sentence. For the filler items, the questions also referred to the content of the target sentence. See Table 1 for example comprehension questions.

#### Procedure

During the experiment, each participant was seated in a dimly lit, sound-proof booth. Stimuli were presented visually on a computer screen placed about 100 cm in front of the participant with yellow letters against a dark blue background. Each trial began with a fixation star that was displayed for 500 ms in the center of the screen and followed by a blank screen for 150 ms. Each stimulus was presented in segments as indicated by the horizontal bars in Table 1. Single word segments were presented for a duration of 350 ms; phrases containing two or three words were presented for 400 or 450 ms, respectively. An interstimulus interval (ISI) of 150 ms was applied between segments. To verify that the participants had read and understood the sentences, each stimulus was followed by a yes/no verification question. After a blank screen of 150 ms, three question marks occurred for 500 ms, followed by the verification question which was presented in its entirety for 4000 ms. Participants were required to respond as quickly and accurately as possible by pressing a “yes” or “no” button on a gamepad. The assignment of the left and right response buttons was counterbalanced across participants. After the question, a blank screen was presented for 400 ms, followed by the next trial. Prior to the experimental run, participants completed a brief practice session to get acquainted with the experimental procedure.

#### EEG recording and preprocessing

The electroencephalogram (EEG) was recorded from 24 Ag/AgCl scalp electrodes and mounted in an elastic cap (*Easycap*, Munich, Germany). Electrode placement adhered to the international 10–20 system. The ground electrode was positioned at AFz. Electrodes were referenced to the left mastoid and re-referenced offline to linked mastoids. To account for artifacts resulting from eye movements, horizontal, and vertical eye movements were monitored by means of two sets of electrode pairs placed at the outer side of each eye for the horizontal electrooculogram (EOG) and above and below the participant's right eye for the vertical EOG. Electrode impedances were kept below 4 kΩ. All EEG and EOG channels were amplified with a *BrainAmp DC amplifier* (Munich, Germany) and digitized with a rate of 500 Hz.

Before averaging, the EEG data were band pass filtered offline with 0.3–20 Hz to remove unsystematic pre-stimulus differences caused by slow signal drifts. This filter has been identified as an appropriate filter for language-related research that overcomes certain drawbacks arising from baseline correction and has been applied by a number of research groups in previous years (e.g., Wolff et al., 2008; Schumacher and Hung, 2012; Kulakova et al., 2014). Next, automatic (set to ±40 μV for the EOG rejection criterion) and manual rejections were performed to exclude trials containing ocular, amplifier saturation, and other artifacts. Trials with incorrect answers or time-outs to the comprehension question were also excluded from the ERP data analysis. The application of all of these rejection criteria amounted to the exclusion of 12.55% of the data points. Average ERPs were time-locked to the onset of the critical pronoun in the target sentence.

#### Data analysis

Statistical analyses were carried out by means of repeated measures analyses of variance (ANOVAs) and were performed with the factors PRONOUN (personal vs. d-pronoun), VERB (TYPE) (active accusative vs. dative experiencer) and CANON(ICITY) (canonical vs. non-canonical). Additionally, REGION OF INTEREST (ROI) entered the analysis as a factor. The analysis was carried out separately for midline and lateral electrode sites. The lateral electrodes were grouped by topographical ROIs which entered the analysis with four levels: left anterior (F3, F7, FC1, FC5), left posterior (CP1, CP5, P3, P7), right anterior (F4, F8, FC2, FC6), right posterior (CP2, CP6, P4, P8). The midline analysis included the six midline sites as levels (Fz, FCz, Cz, CPz, Pz, POz). All statistical analyses were based on the mean amplitude value per condition and were carried out in a hierarchical order. Huynh–Feldt adjustment was applied when the analysis involved factors with more than one degree of freedom in the numerator. The analyses were performed using the ez-package (Lawrence, 2013) in R (R Core Team, 2015).

### Results

**Figure 1:**
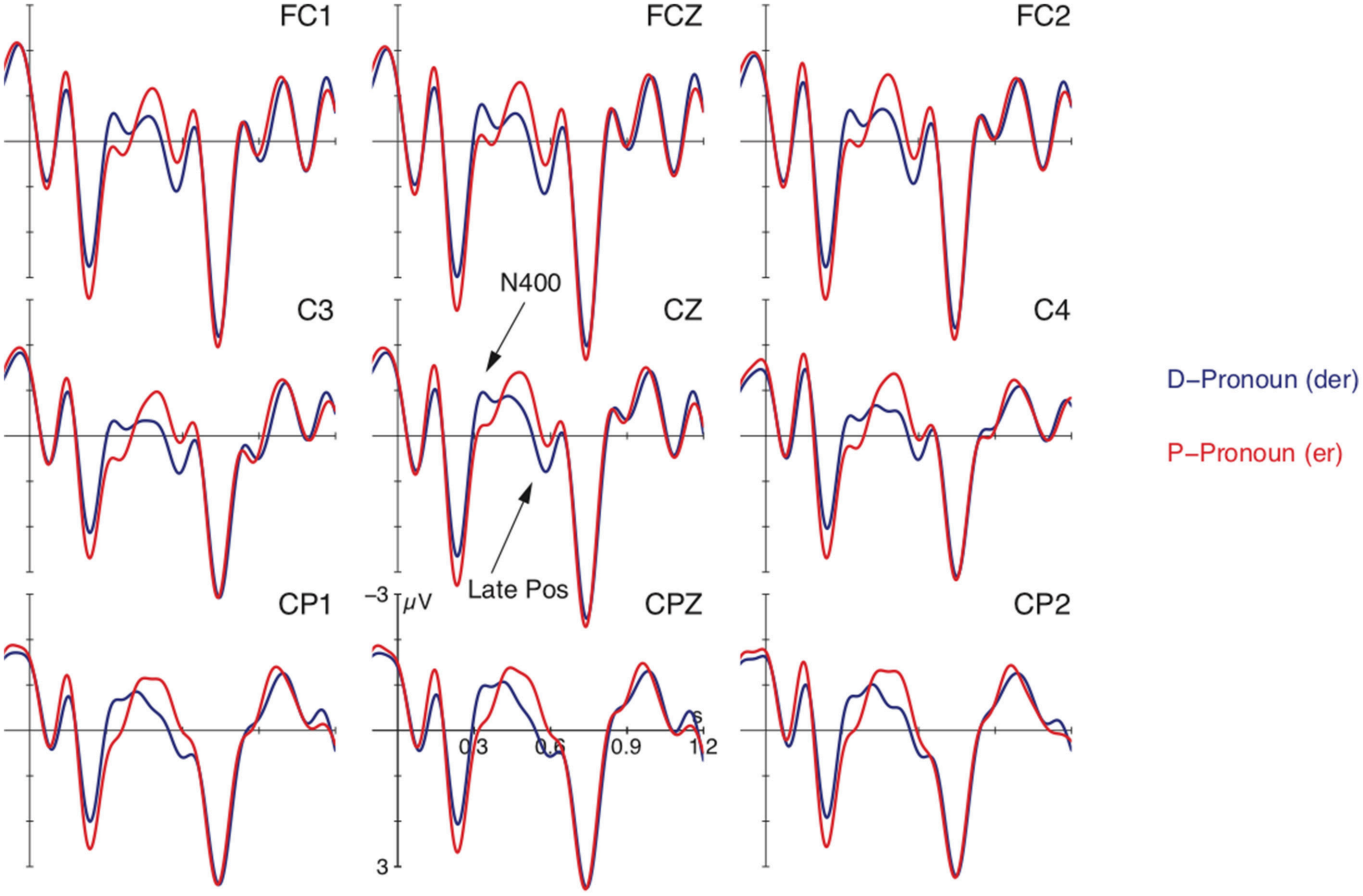
**Grand average ERPs at selected electrodes time-locked to the pronoun for the d-pronoun (blue) and the personal pronoun (red) averaged over both verb types and argument orders**. Pronoun onset is at vertical bar. Negativity is plotted upwards.

**Figure 2:**
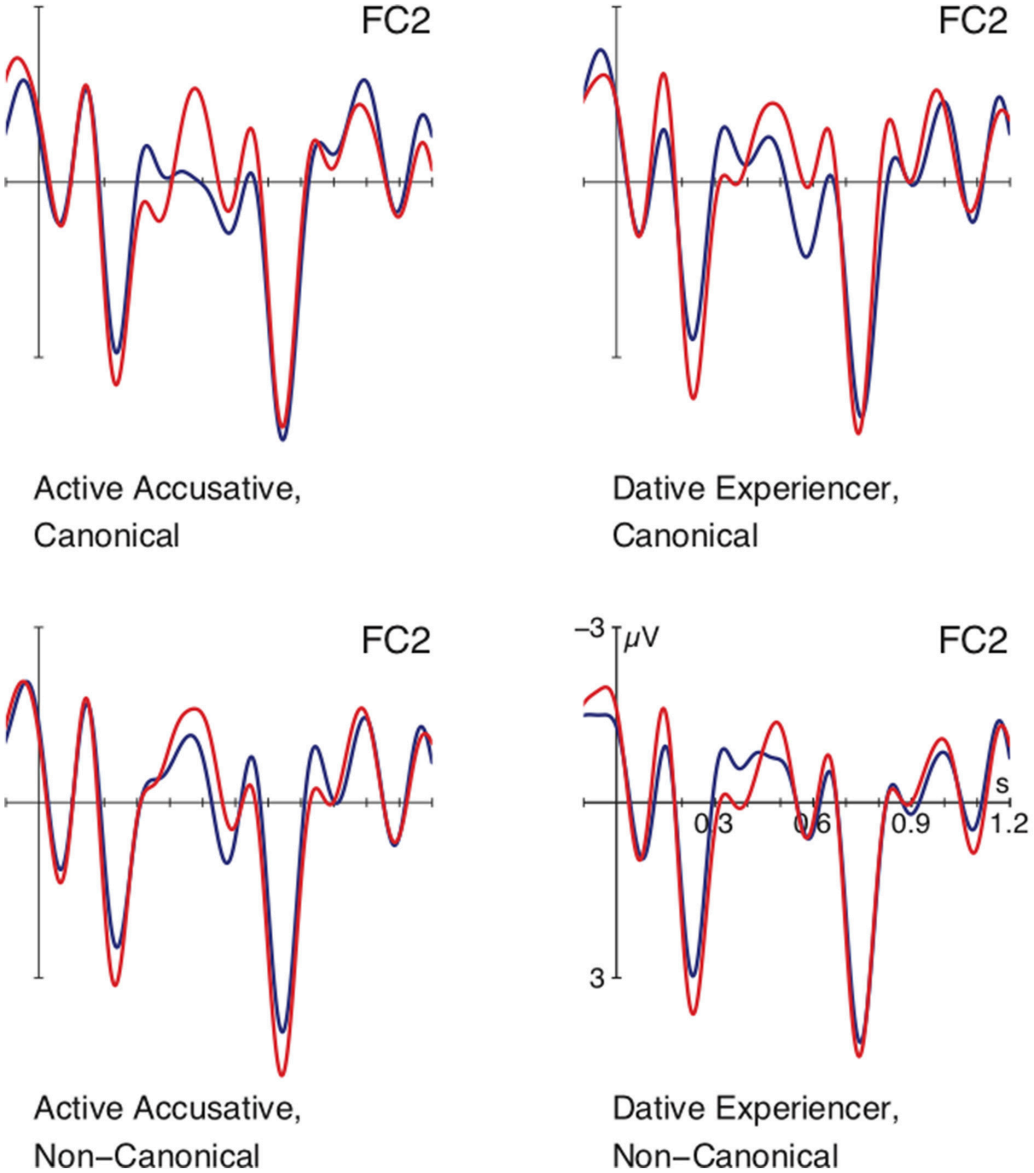
**Grand average ERPs for paired comparisons between the d-pronoun (blue) and the personal pronoun (red) at a selected right anterior electrode site (in which the four-way interaction was resolved)**. Pronoun onset is at vertical bar. Negativity is plotted up.

Figure 1 shows ERPs time-locked to the onset of the personal pronoun (in red) and the d-pronoun (in blue) collapsed over conditions. The plot reveals a negative maximum for the d-pronoun peaking around 300 ms after pronoun onset and a subsequent positive deflection for the d-pronoun between 450 and 600 ms. In addition, there were fine-grained differences arising from the contextual manipulation of verb type and canonicity. This is illustrated by Figure 2 which shows that while the main effect of pronoun—i.e., a negativity around 300 ms followed by a positivity around 500 ms—is found for the two canonical conditions (top row), the non-canonical conditions (bottom row) diverge from the general picture. The non-canonical active accusatives shows no negativity for the d-pronoun over the personal pronoun and the non-canonical dative experiencer contexts seem to have evoked no positivity difference. With the exception of the last comparison, these observations were supported by statistical analyses. After visual inspection, two time windows were determined for the statistical analysis: 275–400 ms for the negativity effect and 450–600 ms for the positivity.

The statistical analysis for the 275–400 ms time window registered a main effect for PRONOUN over lateral electrode sites [*F*_(1, 23)_ = 20.65, *p* < 0.001] as well as over the midline electrodes [*F*_(1, 23)_ = 211.43, *p* < 0.001] and a four-way interaction for PRONOUN × VERB × CANON × ROI [lateral regions: *F*_(3, 69)_ = 3.64, *p* < 0.05; midline electrodes: *F*_(5, 115)_ = 3.86, *p* < 0.05], reflecting the more pronounced negative deflection for the d-pronoun in comparison to the personal pronoun. Separate resolutions of these interactions for lateral and midline regions by region registered no topographical difference for the midline electrodes (and only main effects of PRONOUN over all midline electrodes) but the lateral ROI analysis indicated that the interaction was strongest over right anterior electrode sites [*F*_(1, 23)_ = 4.60, *p* < 0.05]. Subsequent resolution by the factor VERB within this ROI produced the following pattern: for the accusative verbs there was an interaction of PRONOUN × CANON [*F*_(1, 23)_ = 7.31, *p* < 0.01] reflected in an effect of PRONOUN for the canonical subject-before-object order [*F*_(1, 23)_ = 9.24, *p* < 0.01] and no difference between the two types of pronouns in the non-canonical object-before-subject order [*F*_(1, 23)_ < 0.41]. The dative experiencer verbs showed a main effect of PRONOUN [*F*_(1, 23)_ = 4.87, *p* < 0.05] and no interaction of PRONOUN × CANON [*F*_(1, 23)_ < 0.58]. These patterns are illustrated by the pairwise comparisons in Figure 2.

For the time window between 450 and 600 ms, the analyses showed main effects for PRONOUN [lateral sites: *F*_(1, 23)_ = 31.28, *p* < 0.001; midline electrodes: *F*_(1, 23)_ = 23.87, *p* < 0.001] and an interaction of PRONOUN × CANON [lateral: *F*_(1, 23)_ = 8.06, *p* < 0.01; midline: *F*_(1, 23)_ = 4.69, *p* < 0.05]. Resolution of this interaction by CANON showed an effect of PRONOUN for the canonical orders [lateral: *F*_(1, 23)_ = 37.36, *p* < 0.001; midline: *F*_(1, 23)_ = 28.61, *p* < 0.001] and a weaker effect of PRONOUN for the non-canonical orders [*F*_(1, 23)_ = 7.65, *p* < 0.05; midline: *F*_(1, 23)_ = 5.67, *p* < 0.05]. The effects reflect the more enhanced positivity for the d-pronoun over the personal pronoun.

**Figure 3:**
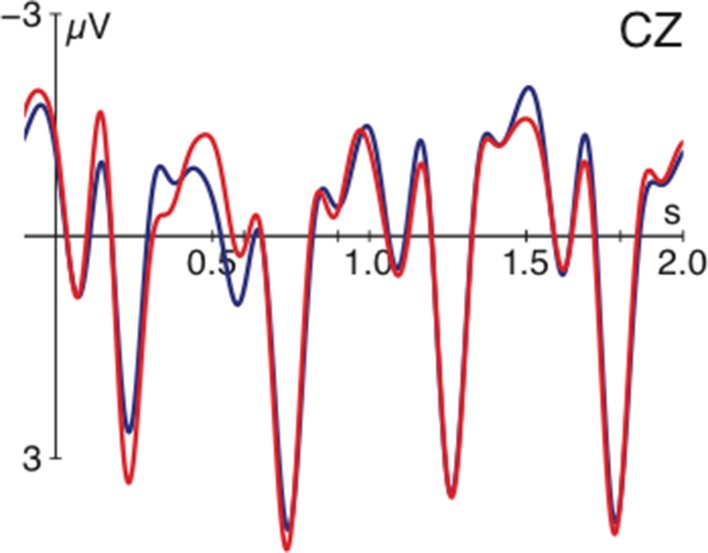
**Grand average ERPs at a selected electrode time-locked to pronoun-onset (at vertical bar) and spanning until 2000 ms later for the d-pronoun (blue) and personal pronoun (red) averaged over the factors verb type and argument order**. Negativity is plotted upwards.

Before turning to the discussion of how these findings inform pronoun resolution, we would like to address one further issue. We want to show that the observed effects for d-pronouns are not due to the ambiguity between the pronoun and the definite determiner in German. According to this, the processing costs registered for the demonstrative could also be caused by the ambiguity between the d-pronoun and the masculine definite determiner (both “der” in German). If costs were due to form ambiguity or anticipation of a noun following the determiner (for a sustained negativity for definite vs. indefinite determiners in German see Schumacher, 2009), additional (reanalysis) processes should be observable in the segment following the critical region. Figure 3 spans until 2000 ms after pronoun onset and illustrates that no effects occurred in spill-over regions. The segment-wise presentation mode chosen in the current investigation may have been conducive to this as well because NPs were always presented in their entirety. We thus exclude form ambiguity as a potential explanation for the observed differences between personal and d-pronouns.

### Discussion

In the discussion of the ERP study, we first focus on the general effects of pronoun in the two time windows before looking at the subtle interactions with the other factors in more detail.

#### Main effect of pronoun

Averaged over canonicity and verb type, the d-pronoun in comparison to the personal pronoun displayed a biphasic pattern with a more pronounced negativity in the early time window between 275 and 400 ms and an enhanced positivity in the later time window between 450 and 600 ms (see Figure 1). We propose that these two effects reflect backward- and forward-looking operations respectively.

The backward-looking function represents a core characteristic of a pronoun, which is referentially deficient and depends on an antecedent. We take the observed negativity (N400) for the d-pronoun as an indication for the more demanding processing of such a dependency relation on the basis of the instruction to exclude the most prominent referential candidate. The N400 for the d-pronoun patterns well with other findings from reference resolution that indicate that more computationally demanding anaphor-antecedent relations engender a negativity, including surface distance, semantic distance or referential ambiguity to name a few (Streb et al., 2004; Burkhardt, 2006; Nieuwland and Van Berkum, 2006). The current data with an enhanced N400 for d-pronouns over personal pronouns add to this view.

An alternative account for the observed cost would be that the d-pronoun is less expected than a personal pronoun (as a result of the information structural topic maintenance preference) and counters the particular prediction for an upcoming referent formed on the basis of prominence structure. Along these lines, the N400 has more generally been described as an expectation-driven process that is enlarged whenever a processing expectation is not met. However, the next section demonstrates that there are subtle interactions of the different prominence-lending cues manipulated in this study. Such findings indicate to us that the N400 for the d-pronoun reflects aspects associated with the prominence structure underlying the set of referential candidates (i.e., backward-looking operations). We assume that coreference relations depend on certain prominence features that govern the ranked set of referential candidates in the mental representation. Coreference with a less prominent entity (assumed for d-pronouns) results in processing costs.

The subsequent positivity (Late Positivity) for the d-pronoun over the personal pronoun is taken to reflect mental model updating costs. While a personal pronoun typically indicates the continuation of the current discourse topic, a d-pronoun signals a possible shift in attention toward a non-topical referent and therefore has a forward-oriented potential in providing cues about the changing (prominence) structure of the upcoming discourse (cf. e.g., Abraham, 2002). The d-pronoun further occurs in the topic position of the target sentence marking an interruption of the referential coherence. The processing of such forward-directed information exerts costs associated with the organization of discourse referents and the maintenance of the mental representation. Previous research on information structural influences on referential processing reported a Late Positivity for topic shift as well as contrastive focus (e.g., Hirotani and Schumacher, 2011; Wang and Schumacher, 2013; Hung and Schumacher, 2014). These information structural phenomena have in common that they can promote the cognitive status of their referents and direct the addressee's attention to a previously less attended referent. Behavioral data substantiate this role of topic and focus constituents (cf. Almor, 1999; Kaiser and Trueswell, 2004; Cowles et al., 2007). For the mental representation this implies that the prominence level of referents may shift dynamically and that any change may result in discourse updating costs. To substantiate these claims and assess whether d-pronouns affect the topic structure of subsequent discourse, we carried out Experiment 2 below.

#### Prominence cues

When we look at the interaction of pronoun type with the two verb types and canonicity, subtle differences occur in particular with respect to processes in the N400 time window. Resolution of the four-way interaction revealed a more pronounced negativity for the d-pronoun over the personal pronoun in all conditions but the non-canonical active accusative antecedent contexts (see Figure 2). We take this to reflect processing differences associated with the computation of prominence, which seems to be most severely encumbered in the latter condition. This is best explained by the alignment based hypothesis (see Table 1): The four antecedent contexts differ with respect to their alignment of a number of potential prominence features, as illustrated by Table 1: (i) proto-agent > proto-patient, (ii) subject > object, and (iii) topic > non-topic (which we take to be a matter of sentence position). In the two canonical argument order cases, in which the proto-agent precedes the proto-patient, the underlying processes look much alike. As Table 1 illustrates, all three prominence-lending cues are aligned to the first argument in the canonical accusative contexts. The canonical dative experiencer contexts differ in that the initial topical argument is the agent but not the subject. This suggests that in this case of partial alignment, the absence of subjecthood does not have a negative impact on computation. And it indicates—in line with previous behavioral data (Schumacher et al., 2016)—that thematic role information represents a more highly ranked constraint during pronoun processing than grammatical function. Yet, grammatical function information still seems to contribute to pronoun comprehension to a certain extent because an N400 difference between personal and d-pronouns is still observed following the non-canonical dative experiencer contexts. In this case, the subject is aligned with the first position. Critically, in the non-canonical conditions, the active accusative condition diverges, which is the condition in which neither thematic role nor grammatical function information is aligned with the initial argument. This constellation apparently has real-time consequences for both personal and d-pronoun comprehension since the N400-morphology of both pronouns following the non-canonical active accusative contexts looks rather different from the other contexts. This suggests to us that the prominence-lending features made available by this particular context are not powerful enough to feed into prominence computation, encumbering coreference dependencies at this point in time.

Prominence computation—i.e., the calculation of a ranked set of referential candidates—thus seems to rely on the combination of weighted constraints over referential candidates. When agent or subject arguments occur in sentence-initial position, the resolution instruction (“corefer with the most prominent referential candidate” for the personal pronoun and “exclude the most prominent referential candidate” for the d-pronoun) can be executed, reflected in more computational demands for the exclusion of a referential candidate in the case of demonstratives. In situations in which the initial position is not aligned with either agent or subject (i.e., the non-canonical active accusative case), processing is hampered for both resolution instructions. This indicates that agents in first position are ideal candidates for referential prominence, regardless of grammatical function. When the first argument does not carry the highest thematic role, subjecthood of this argument enhances its referential status. This also indicates that initial position is one of the crucial cues contributing to referential processing (see e.g., Gernsbacher and Hargreaves, 1988). The first position typically hosts information structurally prominent entities, such as topics in German, which has led to proposals for topic and anti-topic pronominal resolution strategies, adding this feature to the prominence candidate set (Bosch et al., 2007; Hinterwimmer, 2015). One caveat arises from the unlicensed non-canonical argument order utilized in the current context sentences, which might well benefit from a richer context with an established discourse topic that paves the way for a marked argument linearization. In contextually enriched cases, prominence computation in non-canonical active accusative contexts may then be eased after all (cf. the research on information structural influences on argument linearization, e.g., Kaiser and Trueswell, 2004; Schumacher and Hung, 2012; Burmester et al., 2014).

Following our claim that the N400 reflects initial processes of executing the pronoun-specific linking instruction, one might ask how these data connect with the interpretive preferences obtained in previous offline studies (Schumacher et al., 2016). Similar to previous offline data that also tested the factors verb type and canonicity, the statistical analyses indicate more pronounced patterns for the canonical argument order (proto-agent > proto-patient) than for the non-canonical order. Yet the ERP data also differ partially from previous offline data in that the offline measures registered more interpretive insecurity in the non-canonical dative experiencer constructions, while the N400 patterns suggest that the non-canonical active accusative constructions are hampered. Certainly offline preferences may be influenced by additional factors and reflect more conscious and controlled operations. However, the differences between online and offline measures may also point out that the observed N400 effect reflects a more automatic process of prominence computation, which is calculated prior to referent selection^1^. A close look at Figure 2 may even suggest a link between the Late Positivity and the offline data, where the non-canonical dative experiencers showed no positivity for the d-pronoun between 450 and 600 ms. This may be reflected by the Pronoun × Canonicity interaction in this time window, which yielded weaker effects for the two non-canoncial vs. the two canonical orders. However, the hierarchical analysis of the ERP data that we adopted does not allow us to test the non-canonical dative experiencer constructions in isolation. Since the coreference process is a discourse-internal operation, final resolution may well occur within the discourse-updating stage (cf. the two phases of bonding and resolution in e.g., Sanford and Garrod, 1989; Garrod and Terras, 2000). Coreference of personal pronouns is resolved effortlessly because the most prominent entity is maintained, while d-pronouns are more computationally demanding. Misalignments in the earlier prominence computation stage may then result in disruptive processing during discourse updating.

## Experiment 2

In this study we wanted to test, whether d-pronouns have the capacity to initiate a topic shift, which would strengthen our account of the Late Positivity in Experiment 1. We employed a text continuation study, in which participants are provided with context-target sentence pairs and are asked to continue the story by writing six additional sentences. We then determined the topic constituent of each continuation sentence and calculated the topic shift potential of each pronoun, i.e., is the topic of the initial sentence maintained in the story sentences or is the other referent promoted to topic status in subsequent discourse. This ties in with research that previously attested a larger amount of topic shifts for indefinite *this* relative to a regular indefinite NP (cf. Gernsbacher and Shroyer, 1989; Chiriacescu, 2011). Based on the claim that demonstratives are topic shifters (Abraham, 2002), we predict that the d-pronoun should show a higher capacity of topic shifting as the story unfolds, while the personal pronoun should encourage topic maintenance (cf. Grosz et al., 1995 for topic continuity expressed by the personal pronoun). Such a main effect of pronoun would substantiate the claim that the Late Positivity is associated with additional demands due to topic shifting, and based on the findings from Experiment 1 as well as the research literature, we predict more topic shift potential for all d-pronoun conditions irrespective of verb type and canonicity. Note however that there was a pronoun × canonicity interaction in the Late Positivity window in the ERP experiment which resulted from more pronounced effects in the canonical compared to the non-canonical conditions. Accordingly, non-canonical antecedent clauses—and in particular the non-canonical accusative contexts—which show misalignment of topic and agent may impede the dynamic updating of the discourse representation structure.

### Methods

In this survey, participants were presented with context-target sentence pairs and were asked to continue the story by writing down six additional sentences.

#### Participants

Thirty-two native speakers of German (16 women; mean age: 25; range: 18–33 years), all monolingual, from the University of Cologne participated in this online survey. The investigation was performed in accordance with the Declaration of Helsinki and with the national and institutional recommendations of the Empirical Linguistics Lab at the University of Cologne.

#### Materials and procedure

Four active accusative and four dative experiencer constructions were selected from Experiment 1 and each was presented in the four (canonicity × pronoun) versions. To reduce the number of given referents, only the main clause was used from the context sentence, followed by a target sentence with either a personal or a d-pronoun. The 32 critical items were distributed across 16 lists, so that each participant finished two items. Pilot research had shown that presenting more than two continuations is not recommendable.

#### Data analysis

We wanted to find out which referent served as sentence topic in the continuation sentences. To this end we assume that the sentence-initial position holds the sentence topic (cf. aboutness-topic, Reinhart, 1981) and therefore determined whether the initial argument of each continuation reflected a shift or maintenance relative to the story-initial topic.

Each sentence of a continuation was coded with respect to whether it referred to the first or second NP in the context sentence or to another (new) referent that was introduced as part of the continuation. We only analyzed the first five (out of six) continuations, since in this task the last sentence often encourages a summary or wrap-up of the story line. Since we are interested in how the two referents from the initial sentence are picked up in subsequent sentences, reference to newly introduced entities were discarded prior to the analyses. Reference to the initial argument was coded as topic maintenance and reference to the second argument as topic shift. We first calculated the absolute frequency of topic shift and topic maintenance for the eight conditions. We further ran regression analyses with the predictors PRONOUN (personal pronoun; d-pronoun), VERB type (active accusative; dative experiencer) and CANON(ICITY) (canonical; non-canonical).

### Results

**Figure 4:**
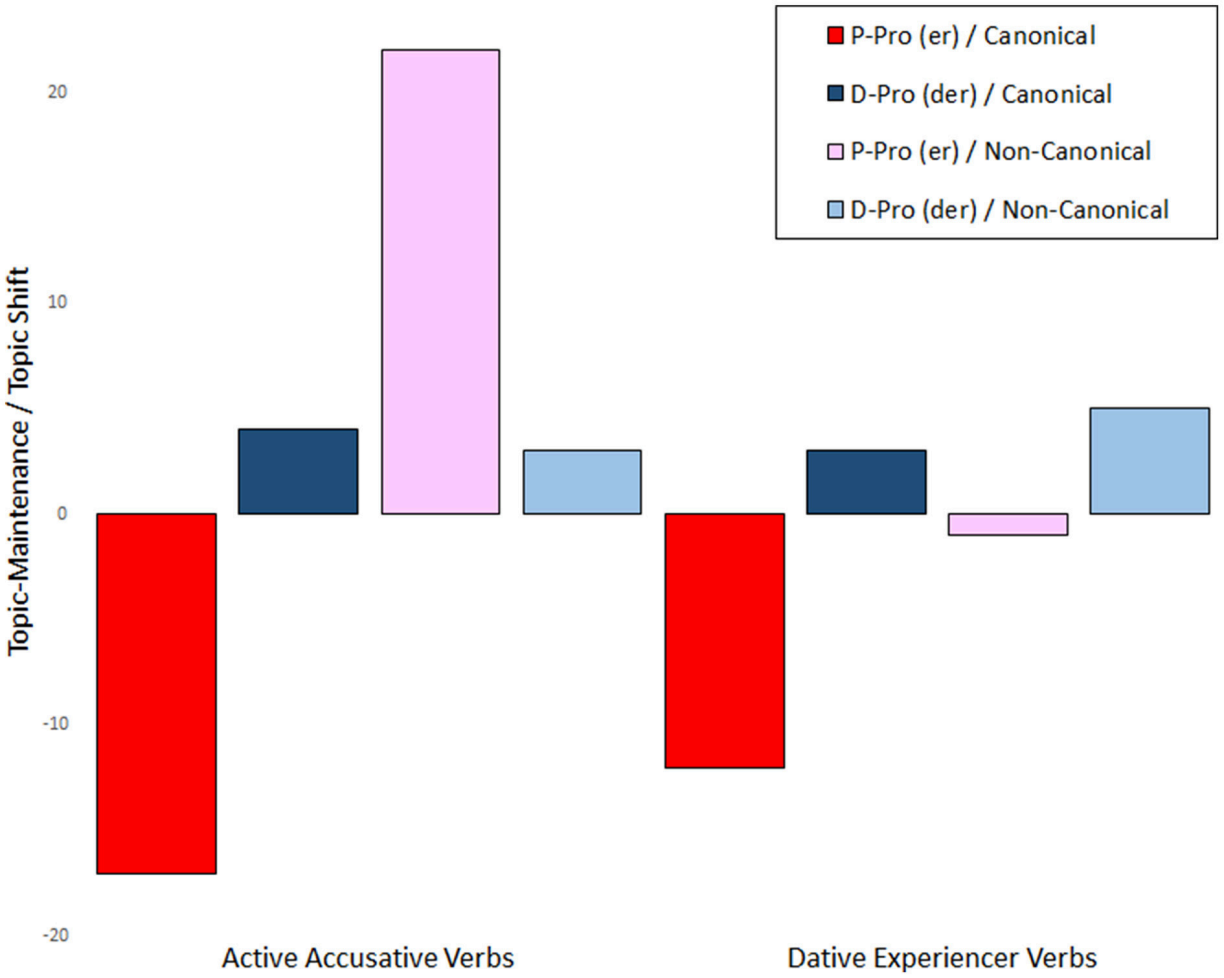
**Forward-directed potential of personal and d-pronouns in the eight conditions**. Preference for topic shift is indicated by positive values (upwards) and for topic maintenance by negative values.

Figure 4 depicts the difference scores determined from subtracting tokens of topic maintenance from tokens of topic shift. It is based on the cumulative absolute frequency of topic maintenance and topic shifts for the eight conditions. Positive values indicate more topic shifts, negative values reflect more topic maintenance. The figure illustrates that personal pronouns (in red) are more likely to maintain the sentence-initial topic—with the exception of the non-canonical active accusative condition—while d-pronouns (in blue) show a small but stable tendency for topic shift.

The regression analysis produced a final model that retained the entire set of effects and interactions. A test of this full model against a model reduced of interactions was statistically significant [likelihood ratio: **χ**^2(4)^ = 20.37, *p* < 0.001]. As predicted the d-pronoun triggered more topic shifts than the personal pronoun. The analysis also showed that non-canonical constructions triggered more topic shifts than their canonical counterparts. As Figure 4 indicates this effect of canonicity as well as the two-way interactions involving canonicity (CANON × PRONOUN and CANON × VERB) and the three-way interaction CANON × VERB × PRONOUN are mainly driven by the unexpected pattern registered for the personal pronoun following the non-canonical active accusative condition. These interactions are reflected by the following patterns: While the d-pronouns show robust topic shift across conditions, personal pronouns in non-canonical antecedent clauses diverge from the topic maintenance observed in the canonical contexts. Active accusative contexts diverge immensely in this regard and even show a large amount of topic shift, while personal pronouns in non-canonical dative experiencer contexts registered only the smallest number of topic maintenance. Table 3 reports the respective coefficients for the topic shift potential with the reference levels “er” for the factor pronoun, “accusative” for verb type and “canonical” for canonicity.

**Table 3:** **Regression analysis of Experiment 2**.

**Predictor**	**Beta**	**SE**	***z***	***p***
Pronoun	1.47	0.55	2.66	0.008
Verb	0.31	0.60	0.53	0.598
Canonicity	3.10	0.69	4.51	0.000
Pronoun ^*^ Verb	-0.37	0.78	-0.48	0.633
Pronoun ^*^ Canonicity	-3.15	0.85	-3.70	0.000
Canonicity ^*^ Verb	-2.26	0.89	-2.55	0.011
Pronoun ^*^ Verb ^*^ Canonicity	2.46	1.14	2.16	0.031

### Discussion

The findings of this text continuation experiment confirm that the different pronouns serve discrete forward-looking functions. They show that the d-pronoun triggers more topic shifts in subsequent discourse than the personal pronoun. This supports previous research on the forward potential of indefinite demonstratives in English and German (cf. Gernsbacher and Shroyer, 1989; Chiriacescu, 2011). The personal pronoun in turn typically prompts topic continuations. The topic shift preference of the d-pronoun corroborates our proposal that the Late Positivity observed in the ERP study is associated with forward-directed signals that are encoded in discourse representation.

Based on these forward-oriented functions, the results for the personal pronoun in the non-canonical antecedent clauses suggest an interplay of prominence computation and discourse updating potential. In particular the pattern observed for the personal pronoun in the non-canonical active accusative constructions is surprising but it also emulates the exceptional role of this condition in Experiment 1, where we argued that the fact that neither proto-agent nor subject are aligned with the first position interferes with prominence computation. This seems to have far reaching consequences for subsequent discourse, where speakers possibly opt for an alternative strategy or even reset their mental representation and pick up the last mentioned referent making this the most prominent one (which results in topic shifts in Experiment 2).

## General discussion

This research supports a dissociation of backward- and forward-looking functions for pronouns and reveals discrete patterns for personal and d-pronouns. The ERP data indicate a discrete time-course of the two functions and the text continuation data strengthen the account that d-pronouns are more likely to initiate a topic shift, while personal pronouns support topic maintenance.

### Backward-looking function

Overall, the current findings call for a resolution algorithm that considers multiple weighted prominence cues. Centering Theory (CT; Grosz et al., 1995) has served as a solid basis for numerous investigations of pronoun resolution. It assumes that certain referents of an utterance are more central than others, which, in turn, affects the processing of the subsequent utterance. Furthermore, personal pronouns are claimed to be preferably resolved toward the most central referential entity, which is understood as a means to establish coherence (Abraham, 2002). Within the CT framework, every utterance may contain several entities that have the potential to establish coherence with the following utterance. These referential expressions are called “Forward-looking Centers” (Cfs) and are ranked according to prominence features, whereby the highest ranked Cf of an utterance is referred to as “Preferred Center” (Cp). To determine if and how coherent two subsequent utterances are, CT offers an algorithm based on two parameters: the cognitive state of the “Backward-looking Center” (Cb), that is the element that picks up the highest ranked Cf from the previous utterance—ideally the Cp—and the current Cb's relation to the Cb of the previous utterance: either the Cb remains the same (Continue or Retain relations) or the Cb changes across two utterances (Smooth or Rough Shift relations; Brennan et al., 1987). Based on pronoun resolution in English, the ranking of the Cfs has been framed according to grammatical function (subject > object > other). Cross-linguistic comparisons however indicate that the setup of prominence cues is subject to language-specific constraints. Research on Japanese and German suggests that information structural notions contribute to the centering algorithm as well which has led to expansion of the grammatical function hierarchy (e.g., for Japanese: topic > empathy > subject > object > other; Kameyama, 1985; Walker et al., 1994, 1998; Di Eugenio, 1998; Abraham, 2002; Speyer, 2007).

While the application of the modified hierarchy may to a certain extent account for utterances with accusative verbs, it does not predict the proto-agent-preference observed for the dative experiencer verbs. We therefore propose to include proto-agentivity as a high-ranking constraint for the Cf ordering in German (proto-agent > proto-recipient > proto-patient; cf. e.g., Dowty, 1991; Primus, 1999). This shift from the grammatical function to the thematic role hierarchy does not affect the results for the canonical sentences with accusative verbs since the highest Cf is also the subject, but it serves to explain the preferences observed for the dative experiencer verbs in which subject and agent are assigned to distinct referents. Due to the non-canonical linearizations, we further suggest to consider information structural notions as suggested previously on the basis of data from German and Japanese (cf. e.g., Walker et al., 1994; Abraham, 2002; Speyer, 2007). In particular, positional cues in the antecedent clause mark additional information status, with initial entities signaling topic status or contrast. In our case this information structural function may be weakened by the contextually unmotivated placement of a discourse-new object in initial position of the context sentence. But nevertheless first position in combination with other prominence-lending cues provides important information for prominence computation. The current data thus suggest an intricate interaction of agentivity, information structure, and subjecthood, which needs to be tested in more elaborate discourse contexts in future research. Furthermore, CT typically considers only the set of Cfs from the previous utterance; yet, larger discourse structure should be incorporated into CT algorithms. To summarize the backward-looking processes, the data indicate that the thematic role cue is tied to positional information, i.e., agents in initial position are the best candidates for prominence in the current study. In cases, where agents are not aligned with the initial position, grammatical function information collaborates with positional information to boost referential prominence.

Finally, a CT-like algorithm should also account for the resolution of demonstratives. In particular, resolution processes should exclude the Cp as a potential antecedent for the d-pronoun. In the absence of evidence to the contrary, this assumes that personal and d-pronouns in German make use of the same constraints over prominence structure (contra Kaiser's claims of form-specific constraints in Finnish, see Kaiser and Trueswell, 2008). In this regard, the Cp holds an important function within the referential space, which singles it out from the set of referential candidates.

### Forward-looking function

While demonstratives have been described as topic shifters, this forward-directed potential of referential expressions has been neglected in the research literature to a large extent (with the notable exceptions of Gernsbacher and Shroyer, 1989; Chiriacescu, 2011). To our knowledge, the continuation data from Experiment 2 represent the first test of the predictive potential of d-pronouns. They show that personal and d-pronouns influence the structure of subsequent discourse in different ways, yielding more topic maintenance and more topic shift respectively. This forward function of the pronouns can be regarded as a signal-driven cue whereby the d-pronoun promotes attention reorienting toward a new topic.

This finding thus strengthens our account of the Late Positivity in Experiment 1 as a marker of mental model updating triggered by the d-pronoun's inherent instruction to change the overall topic structure. Based on previous ERP research, we predicted a positive deflection for topic shift and attention orienting more generally, which is supported by the main effect of pronoun in the later time window with a more pronounced positive deflection for the d-pronoun relative to the personal pronoun. This suggests that the forward-looking function has real-time consequences during processing.

As far as the difficulties with the non-canonical active accusative contexts are concerned, the behavioral and ERP data converge. While the online data show no difference between the two pronouns in the N400 window—in contrast to all the other conditions—which we attributed to weak cues for prominence computation, this condition also diverges for the discourse continuation behavior by showing a surprising topic shift preference. This suggests that forward-oriented processing may be affected by the prominence structure of the preceding discourse. In the case where neither agent nor subject align with the first position, the relevant ranking of the referents seems to be destabilized hampering the typical forward potential of the personal pronoun.

## Conclusion

The current investigation revealed differences in the time course of the resolution of personal and d-pronouns, reflected by a biphasic N400—Late Positivity pattern. We suggest that the N400 effect manifests an automatic operation of prominence computation that feeds into the pronoun-specific resolution instruction (“corefer with vs. exclude the most prominent referential candidate”). This early process is further influenced by verb specific information and word order, where the cooccurrence of agentivity and initial position yields an ideal candidate for referential prominence in German but prominence calculation may also be aggravated when particular prominence-lending cues are not aligned. The Late Positivity displays a discourse-internal updating process that provides cues for the possible change in prominence structure of the upcoming discourse, which is also supported by the story continuation task.

### Conflict of interest statement

The authors declare that the research was conducted in the absence of any commercial or financial relationships that could be construed as a potential conflict of interest.

## Footnotes

^1^The ERP data may also be informative for a debate in the theoretical literature about the status of the dative experiencer linearizations. While we followed Haider (1993) among others who takes the dative-nominative order to be canonical, Barddal et al. (2014) argue that the two available argument orders alternate because both arguments carry certain subject features. This claim is supported by patterns of subject-verb inversion, covert realizations in control infinitives or reflexivization. While previous behavioral data (with uncertainty in the case of the nominative-dative order) did not strengthen this latter view, the N400 data show no order difference for the dative experiencer constructions.
